# Ultra High-efficiency Integrated Mid Infrared to Visible Up-conversion System

**DOI:** 10.1038/s41598-020-66392-0

**Published:** 2020-06-09

**Authors:** Aytak Motmaen, Ali Rostami, Samiye Matloub

**Affiliations:** 1 Photonics and Nanocrystals Research Lab (PNRL), University of Tabriz, Tabriz, 5166614761 Iran; 2 SP-EPT Lab., ASEPE Company, Industrial Park of Advanced Technologies, Tabriz, 5364196795 Iran; 3 Quantum Photonics Research Lab (QPRL), University of Tabriz, Tabriz, 5166614761 Iran

**Keywords:** Optoelectronic devices and components, Quantum dots

## Abstract

In this paper, we have introduced and investigated an integrated optoelectronic chip for the up-conversion of mid-infrared to visible light. A thin layer of the nanocrystalline photoconductive PbSe is put on the Base of the NPN bipolar junction transistor and a doped phosphorescence organic light-emitting diode is placed on the Collector contacts. The incoming mid-infrared light is converted into an electric current by quantum dot photodetector, then amplified by the NPN bipolar junction transistor, and finally, the amplified current is driven through the Collector in the organic light-emitting diode. The organic light-emitting diode is designed to emit a green color. Our findings indicated that the proposed devices provide an up-conversion process from mid-infrared to visible light with a high-efficiency rate. The quantum dot photodetector is designed to detect 3 μm and also the organic light-emitting diode works at 523 nm. It is easy to tune the 3 ~ 5 μm incoming light by tuning the PbSe quantum dots, and the output light is tuned by tuning the organic light-emitting diode structure. Thus, the proposed structure is highly flexible regarding receiving mid-infrared and generating visible light. It is concluded that the external quantum efficiency for the proposed structure for 3 μm to 523 nm is 600. Also, the enhancement of the transistor current gain (β) can further increase the conversion efficiency of the proposed device. Moreover, different structures such as Darlington can be used instead of the bipolar junction transistor to enhance conversion efficiency.

## Introduction

Up-conversion devices have attracted much interest because of their applications in many fields of science & industry, such as night vision, homeland security, range finding, telecommunications, and semiconductor wafer inspection as well as medical imaging^1–7^. Commercial near-infrared (NIR) imagers are fabricated by integrating III-V compound semiconductor photodetectors (PDs) with silicon read-out integrated circuits (ROIC) using the indium bump bonding technology^8–10^. These typical III-V compound semiconductors (such as InGaAs) have a spectral response ranging from 1 μm to 1.8 μm, so these NIR imagers can not detect NIR as well as visible images^11,12^. Furthermore, the indium bump bonding process increases the fabrication costs of these imaging devices, so they are not always economical.

Long-wavelength to short-wavelength up-converter devices have been indicated in the last two decades as an alternative technology for infrared (IR) imaging^13–15^. In recent years, Liu *et al*. introduced a NIR to visible light up-conversion device by integrating a light-emitting diode (LED) with an IR photodetector (IRPD)^13,16–19^. For example, the NIR up-converter, which had been fabricated by growing InGaAs/InP PD and InAsP/InP LED on InP substrate in 2000, had a low-efficiency up-conversion rate (˂0.0005 W/W) due to the low quantum efficiency (QE) of the LED^13^. The GaAs/AlGaAs LED had a higher efficiency and could be utilized instead of the InAsP/InP LED. Besides, it is difficult to match the lattice between the PD and the LED. To solve this problem, the PDs and LEDs have been bonded through-wafer fusion. This method resulted in increasing efficiency (~0.018 W/W)^17^, but the fusion wafer process technique has its limitations and challenges that make it less attractive^20^.

The next process of the inorganic NIR up-conversion device was the growth of both the PD and LED on the inorganic substrate via epitaxial growing^16^. Although this up-conversion had a higher QE than the previous method (0.048 W/W), the epitaxial growth of inorganic up-conversion devices are costly^16^. In general, they are not economical because of their low efficiency and limited range of up-conversion from 1.5 μm to 1 μm. Then, the optoelectronic device based on organic material was realized by integrating an Organic-LED (OLED) with an organic PD^21,22^. Yase *et al*. reported the fluorescent OLEDs with titanyl phthalocyanine (TiOPc) that exhibited NIR-to-blue and red-to-green up-conversion. These up-conversion devices showed a QE less than 0.05% and had a lower external quantum efficiency (EQE) due to the low efficiency in the OLEDs and the organic PD. But more recently, Kim *et al*. reported all-organic NIR-to-visible up-conversion devices with an SnPc: C_60_ layer as NIR sensitizer and a CBP(Irppy_3_) layer as a phosphorescent emitter^23^. This up-conversion device has a higher QE (2.7%W/W) than the previous methods due to the high efficiency of the OLED and PD^24,25^. These devices are not sensitive to more than 1 μm^23^.

To detect photons at long wavelengths, Liu *et al*. reported inorganic semiconductors featuring a low band gap^26,27^. They reported a hybrid organic/inorganic up-conversion device that was made by the integration of an inorganic InGaAs/InP PD with an OLED^26,27^. In this structure, the QE for 1.5 μm IR light illumination (1.1 mW/cm^2^) was only 0.7%^26^. Chen *et al*. proposed a hybrid optical up-conversion by integrating a heterojunction phototransistor and an OLED^28,29^ to improve the efficiency of up-conversion devices that had been fabricated by Liu *et al*.^26,27^. In this structure, the QE for 1.5 μm IR light illumination (1.2 mW/cm^2^) was 1.55 W/W. In general, they are not economical because of their low efficiency and the epitaxial growth of inorganic semiconductor material. Also, these hybrid up-conversion devices are not sensitive to ranges higher than 1.5 μm.

Kim *et al*. fabricated hybrid up-conversion devices by integrating a colloidal PbSe nanocrystal NIR layer on the OLED^30^. The PbSe nanocrystals were chosen because of the absorption of 0.7 μm to 2 μm in this structure^31–33^. A ZnO nanocrystal was used as the blocking layer of holes between the ITO anode and the PbSe layer to keep the device off without NIR light radiation. The maximum external conversion efficiency was 1.3% and 1.3 μm to 0.52 μm for the IR light conversion. Kim *et al*. introduced an imaging system that is capable of capturing images at NIR as well as for visible wavelengths^34^. This imaging system includes a digital single-lens reflex camera and an IR sensitive OLED. The IR-OLED with the PbS nanocrystals IR sensitizing layer captures IR images with a wavelength of 1.2 μm and converts them into visible images. An IR-pass-visible mirror is inserted between the glass substrate and the ITO anode to transmit the IR and reflect the visible light. It also increases the transparency of the IR-OLED. The maximum external conversion efficiency for the 1.2 μm wavelength is 1.02%. However, the EQE reported by Kim^30,34^ are very low. These up-conversion devices have two terminals, and the electric fields in the OLED and the PD cannot be separately controlled, so the connection of these two parts with elements featuring a higher efficiency does not necessarily lead to higher efficiency in the up-conversion device^30,34^.

Recently, high-efficiency IRPDs based on colloidal quantum dot (QD), graphene and organic semiconductors with gain have been reported^35–38^. The gain in these devices is due to the carrier mobility and so responsivity is slow. Consequently, Yu *et al*. reported IR-to-visible up-conversion devices that integrate a high-gain vertical phototransistor with a perforated metallic source electrode and a phosphorescent OLED^39^. The three-terminal phototransistor contains an IR photo-active gate. Furthermore, the photoactive gate consists of a solution-processed QD (PbS) layer. The Up-conversion photon-to-photon conversion efficiency for a low IR illumination (λ = 1.043 μm, 2.54 μW/cm^2^) was 1000% and the photon-to-photon conversion efficiency was approximately 20% for a light intensity of 75 μW/cm^2^. To improve efficiency, Li *et al*. have fabricated a NIR absorption layer that incorporates between the carrier transport layer and the emission layer in heterostructured organic field-effect transistors^40^. The photon-to-photon conversion efficiency was 28.7% for a NIR light intensity of 10.4 μW/cm^2^ and 0.93% for 196 mW/cm^2^. Due to the device layout, it is probably less suitable for large-area direct imaging applications.

Organic optoelectronic devices have been actively studied in the past years due to their advantages such as flexibility, low processing-cost, simple fabrication techniques, and wavelength selectivily^41–46^. Recently, quantum dot lasers (QDLs), quantum dot semiconductor optical amplifiers (QDSOAs) and PDs have been developed by using of solution-processed^47–51^. Zhang *et al*. reported solution-processed organic PDs (OPDs) with a photomultiplication (PM) effect^50,51^. The device structure of single-layered PM-OPD is designed with an active layer for electron tunneling injection that can work under forward and reverse bias. This PM-OPD exhibits the maximum EQE value of 3900% at 355 nm wavelength and 4900% at 640 nm wavelength and the maximum specific detectivity (D^*^) of 3.2 × 10^12^ Jones and 6.5 × 10^12^ Jones under a 5 V and -5V bias, respectively^50^. Also, Zhang’s group was designed double-layered PM-OPD with one absorber and one multiplication layer^51^. The response range of the double-layered PM-OPD is 350 nm to 950 nm. The EQE values increase along with an increase in voltage, and the largest EQE value is 200% and 1200% under a 2 V and 10 V bias, respectively. Also, the detectivity is 6.8 × 10^12^ Jones and 6.8 × 10^12^ Jones for 350 nm and 950 nm wavelength, respectively.

In this research work, we have designed and developed an up-conversion device by working on the mid-infrared (MIR) light range ($3\, \sim \,5\,{\rm{\mu }}{\rm{m}}$) and the improvement of the EQE. This up-conversion device includes a doped nanocrystalline layer of PbSe as a photoconductive PD, a NPN (n-type, p-type, n-type) Bipolar Junction Transistor (NPN BJT) as a current amplifier, and an OLED. The fabricated up-conversion device has three terminals and an electrical field in the OLED and the PD, which can be separately controlled. The calculated optical power efficiency for a light intensity of 0.5 mW/cm^2^ under the OLED voltage of 10 V and the PD voltage of 6 mV is 600.

## Methods and Materials

**Figure 1:**
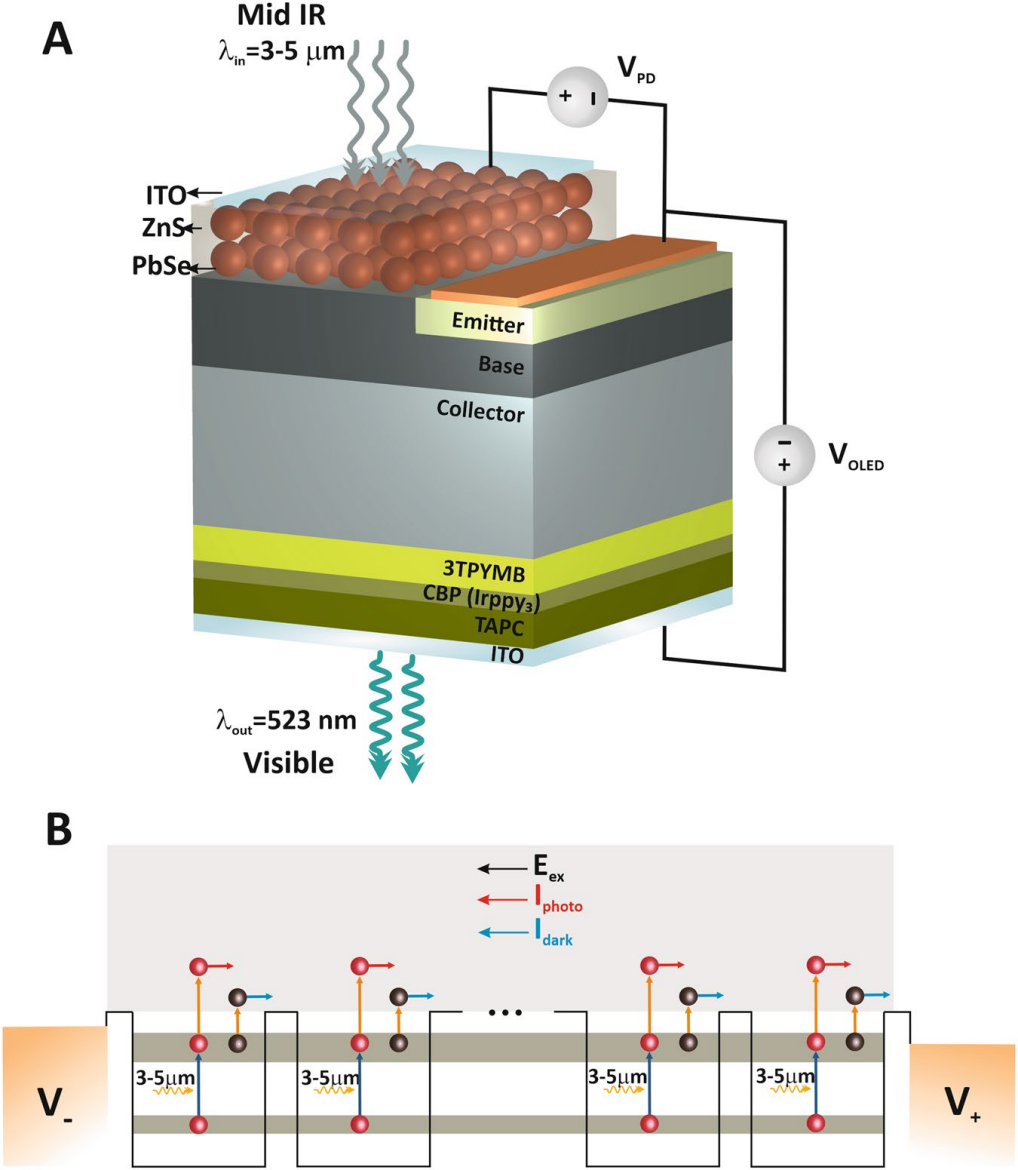
Schematic illustration of MIR-to-Visible light upconversion device. (**A**) Schematic illustration of MIR-to-Visible light upconversion device, (**B**) Schematic representation of energy band diagram of the PbSe/ZnS QDPD and intersubband transition, the transfer of electrons from energy levels of ES to continuum using environment thermal energy causes to a dark current (blak circle, Idark) and photons excite electrons from energy levels of GS to energy levels of ES, causing a photocurrent (red circle, Iphoto).

In this paper, we plan the MIR-to-visible light up-conversion structure that integrates with a thin layer nanocrystalline PbSe as a PD, the NPN-BJT as a current amplifier, and the OLED as a visible light generator on a compact device. For current amplification, a standard NPN-BJT with a given gain is considered (β = 100). For the OLED part of the structure, a TAPC layer as a hole transporting layer (HTL), a CBP layer doped with Irppy3 as an emitting layer and 3TPYBM as an electron transporting (hole blocker) layer (ETL) are used. The nanocrystalline PbSe can absorb incoming MIR light and generate photo carriers that are amplified by a Transistor and then it is injected into the OLED to emit visible light (green) that is visible to the normal vision. A schematic illustration of the proposed MIR-to-visible light up-conversion structure is shown in Fig. 1(A). As shown in this figure, the MIR absorption part is a thin layer of PbSe QDs that grown on the substrate ZnS and this layer connected to the Transistor base. The PbSe quantum dot photodetector **(**QDPD) is passivated by a transparent conductor to remove the interaction between nanoparticles and environment charges and materials. MIR light is absorbed by the QDPD, producing electron-hole pairs. These photo-generated electrons in the QDPD are amplified by a Transistor and collected in the collector region. Because a forward bias is applied to the OLED, the electrons inject to the OLED ETL layer from the collector, and the holes inject the OLED HTL layer from ITO anode. Then the recombination of electrons and holes in the emission region of the OLED results in the production of green light.

By considering the presented schematics, it is easy to say that in this structure, MIR wavelength is converted to the visible wavelength. Also, it is flexible to convert any wavelength in MIR range to standard visible wavelengths. PbSe QDs is used for long wavelengths detection, which has high optical absorption and the narrow bandgap that can be adjusted to 3~5 μm. The QDPD can absorb any wavelength in the MIR region by changing the QDs size. The radius of PbSe QD for the absorption peak of 3 μm is 2.58 nm.

The Poisson and Schrödinger equations have been used self to consistently calculated the eigenenergy, corresponding eigenfunction, electron concentrations of each level, and the potential profile of a PbSe/ZnS QDs structure. So these elements are an important requirement to analyze the proposed structure. The one-dimensional Schrödinger equation at PbSe/ZnS QDs structure can be written by^52–55^:

1 $$\frac{-{\hslash }^{2}}{2{m}_{e}^{\ast }}\frac{{d}^{2}}{d{x}^{2}}{\psi }_{n}(x)+V(x){\psi }_{n}(x)={E}_{n}{\psi }_{n}(x)$$

where $\hslash$ is the reduced plank constant, ${m}_{e}^{\ast }$ is the effective mass of the electron, ${\psi }_{n}(x)$ is the eigenfunction of electrons in the ground state (GS) and excited state (ES) (*n* = GS or ES) with energy *E*_*n*_, and *V*(*x*) is obtained from the conduction band of the structure, which is determined by the band offsets at the material interfaces combined with the electrostatic potential from the Poisson equation. The one- dimensional Poisson equation is:

2 $$\frac{d}{dx}\left({\varepsilon }_{s}(x)\frac{d}{dx}\right)\varphi (x)=\frac{-q({N}_{D}(x)-{N}_{n,i}-{N}_{A}(x))}{{\varepsilon }_{0}}$$

where ${\varepsilon }_{s}$ is material dielectric constant, ${\varepsilon }_{0}$ is the permittivity of vacuum, $\varphi$ is the electrostatic potential, ${N}_{D}$ is the ionized donor concentration and $\,{N}_{A}$ is the ionized acceptor’s density. ${N}_{n,i}$ is the number of electrons per unit volume in the i^th^ energy levels of GS (ES) that is given by:

3 $${N}_{n,i}=\frac{kT{m}_{e}^{\ast }}{\pi {\hslash }^{2}}\sum _{n}\varTheta ({E}_{F}-{E}_{n})\mathrm{ln}\left[1+exp\left(\frac{{E}_{F}-{E}_{n}}{kT}\right)\right]{|{\psi }_{n}|}^{2}$$

where *E*_*F*_, *E*_*n*_, and $\varTheta$ are the Fermi level, the eigenenergy states corresponding to the GS (ES), and the Heaviside step function, respectively. In the following, the methods of calculation of the detection parameters such as dark current, photocurrent, the responsivity, noise response, and the detectivity have been presented. The photocurrent can be generated as a result of photoexcitation in QDs due to the electron transitions between inter sub-bands in the conduction band when the QDPD is under MIR illumination, as shown in Fig. 1(B). The photocurrent is considered and given by^56^:

4 $${I}_{{\rm{photo}}}=q\phi {\eta }_{{\rm{\alpha }}}{g}_{{\rm{photo}}}$$

where q, $\phi$ and ${\eta }_{{\rm{\alpha }}}$ are the electric charge, the incident number of photons per second, and QE, respectively. Also *g*_photo_ is the optical gain of the photoconductive device. The optical gain states that a single photon can be produced from more than one carrier. In other words, the optical gain can express the total rate of excited carriers. If the lifetime of the carriers is longer than the carrier’s transit time, the gain will be higher than one. The optical gain in QDPD is given by the following equation^56,57^:

5 $${g}_{{\rm{photo}}}=\frac{{\tau }_{{\rm{life}}}}{{\tau }_{{\rm{trans}}}}$$

where ${\tau }_{{\rm{life}}}$ and ${\tau }_{{\rm{trans}}}$ are lifetime and transit time, respectively. ${\tau }_{{\rm{trans}}}$ is the transit time for an electron across one QD region or the period of the structure that obtained by^57,58^:

6 $${\tau }_{{\rm{trans}}}=\frac{{L}_{{\rm{x}}}}{\mu  {\mathcal E} }$$

where ${L}_{{\rm{x}}}$ is the length of the device, μ is the mobility, $\, {\mathcal E}$ is the electrical field. Also in the following, the QE, η_α_, in the length of the device ${L}_{{\rm{x}}}$ given by^56^:

7 $${\eta }_{{\rm{\alpha }}}=(1-{e}^{-\alpha {L}_{{\rm{x}}}})$$

where α is the total intersubband absorption coefficient. The energy levels of QDs are broadened as a result of the size fluctuation of QDs during the fabrication process, which is considered as inhomogeneous broadening (IHB) modeled by the Gaussian distribution function. Therefore, the absorption coefficient is the sum of all intersubband absorption between the broadened energy levels given by^59,60^:

8 $$\alpha =\frac{E}{c{n}_{r}{\varepsilon }_{0}\hslash }\sum _{i}{|{\mu }_{{\rm{EG}},i}|}^{2}({N}_{{\rm{GS}},i}-{N}_{ES,i})G(E-{E}_{{\rm{EG}}}) {\mathcal L} ({E}_{{\rm{ES}},i}-{E}_{{\rm{GS}},i}-E)$$

where ${n}_{r}$ and $c$ are refractive index and speed of light in vacuum, respectively, and $E$ is the incident photon energy. ${E}_{GS,i}$ (${E}_{ES,i}$) and ${N}_{GS}$ (${N}_{ES}$) are the i^th^ energy levels of GS (ES) and the number of electrons per unit volume in the i^th^ energy levels of GS (ES), respectively. Also, ${\mu }_{EG,i}$ is the intersubband optical dipole moment between the initial (GS) and final (ES) states which is given by:

9 $${\mu }_{{\rm{EG}},i}=\iint {\psi }_{{\rm{ES}}}^{\ast }q(x\hat{x}+y\hat{y}){\psi }_{{\rm{GS}}}dxdy$$

where ${\psi }_{{\rm{GS}}}$ and ${\psi }_{{\rm{ES}}}$ are the corresponding eigenfunction of GS and ES, respectively. The IHB of energy levels can be modeled by the Gaussian function around the central interband transition energy, ${E}_{{\rm{EG}}}={E}_{{\rm{ES}},{\rm{central}}}-{E}_{{\rm{GS}},{\rm{central}}}$ which is defined as:

10 $$G(E-{E}_{{\rm{EG}}})=\frac{1}{\sqrt{2\pi }{\xi }_{0}}\exp \left(\frac{-{(E-{E}_{{\rm{EG}}})}^{2}}{2{\xi }_{0}^{2}}\right)$$

The FWHW of IHB equal to ${\varGamma }_{0}=2\sqrt{2Ln2}{\xi }_{0}\approx 2.35{\xi }_{0}$. The Lorentzian function ${\mathcal L}$ with a linewidth of $2\gamma$ has been considered to account for homogenous broadening caused by carrier scattering process.:

11 $$ {\mathcal L} ({E}_{{\rm{ES}}}-{E}_{{\rm{GS}}}-E)=\frac{\frac{\gamma }{\pi }}{{({E}_{{\rm{ES}}}-{E}_{{\rm{GS}}}-E)}^{2}+{\gamma }^{2}}$$

where the factor $\pi$ has been included in a way that the area under the Lorentzian function is properly normalized. The small electric current flows through the PD in the absence of incident radiation known as the dark current is calculated as^60^:

12 $${I}_{{\rm{dark}}}=A\mathop{\sum }\limits_{i=1}^{n}qv{N}_{{{\rm{ES}}}_{i}}\exp \left(-\frac{{E}_{{\rm{a}}}}{KT}\right)$$

where T, K, A, and υ are temperature, Boltzmann constant, PD area, and the electron drift velocity, respectively. E_a_ is the thermal activation energy, which equals the energy difference between the top of the barrier and the energy-related to the last mini-band in the QD. The sensitivity of QDPD can be quantified by considering responsivity and noise performance that determines the minimum level of optical power that a detector can distinguish. The responsivity is the ratio of output electric current to optical input that is measured in A/W and it is given by^56^:

13 $$ {\mathcal R} =\frac{{I}_{{\rm{photo}}}}{{P}_{in}}$$

where ${P}_{in}$ is input optical power. Another critical parameter used to delineate PDs performance is specific detectivity (D^*^). Specific detectivity provides both of the dark current density and responsivity of a device and compares the amount of signal current generation for a given amount of noise at a specific wavelength. The specific detectivity is expressed in units of Jones (${\rm{cm}}\sqrt{{\rm{Hz}}}/{\rm{W}}$) and it is given by^56,61^:

14 $${D}^{\ast }=\frac{{ {\mathcal R} }_{P}\sqrt{A\Delta f}}{{i}_{n}}$$

where ${ {\mathcal R} }_{P}$ is the peak responsivity and $\Delta f$ is the bandwidth and *i*_*n*_ is the noise current^58^. The total noise for a PD can be given as a sum of generation-recombination (shot) and the Johnson (thermal) noise terms. In the designed QDPD the motion of the electron is thermal, so the dominant noise is the Johnson noise. The current noise spectral density is calculated by^56^:

15 $${i}_{n}=\sqrt{\frac{4kT\Delta f{I}_{dark}}{{V}_{PD}}}$$

## Simulation and Discussion

We numerically investigated the proposed up-conversion system. With this aim and hypothesis, a doped thin layer of PbSe QDs is grown on the ZnS substrate, as a PD is used(N-type doped with 5 × 10^18^ 1/cm^2^). The parameters used for the simulation of the PD are given in Table 1. The eigenenergy, corresponding eigenfunction, electron concentrations of each level, and the potential profile of PbSe/ZnS QDs are obtained from solution Eqs. 1–3. Using the calculated electron concentrations of each level, the parameters presented in Eqs. 4–15 can be easily calculated for the PbSe QDPD. Figure 1(B) shows schematically the energy band diagram of the PbSe/ZnS QDPD with the energy mini-bands and electrons intersubband transition. The average distance between the low mini-band and the high mini-band is 0.41 eV, which will lead to the detection of light at a wavelength of 3 μm.

**Table 1:** Figures of merit parameters for the PbSe/ZnS QDPD.

Parameter	Value
The radius of QD (R)	2.58 [nm]
Dot-to-Dot separation	1.6 [nm]
${m}_{e}^{\ast }$ (PbSe)	0.04 m_0_
${m}_{e}^{\ast }$ (ZnS)	0.22 m_0_
The dielectric constant of PbSe (ε_r_)	23.6
The dielectric constant of ZnS (ε_r_)	8.6
Electron affinity of PbSe (χ)	4.5 [eV]
Electron affinity of ZnS (χ)	3.8 [eV]
Bandgap of PbSe (E_g_)	0.278 [eV]
Bandgap of ZnS (E_g_)	3.715 [eV]
electron lifetime of PbSe (τ_r_)^62^	40 [ns]
electron Velocity of PbSe (υ_d_)	1050 m/s
Temperature (T)	300 K
Dopping of PbSe (N_D_)	5 × 10^18^ 1/cm^3^

**Figure 2:**
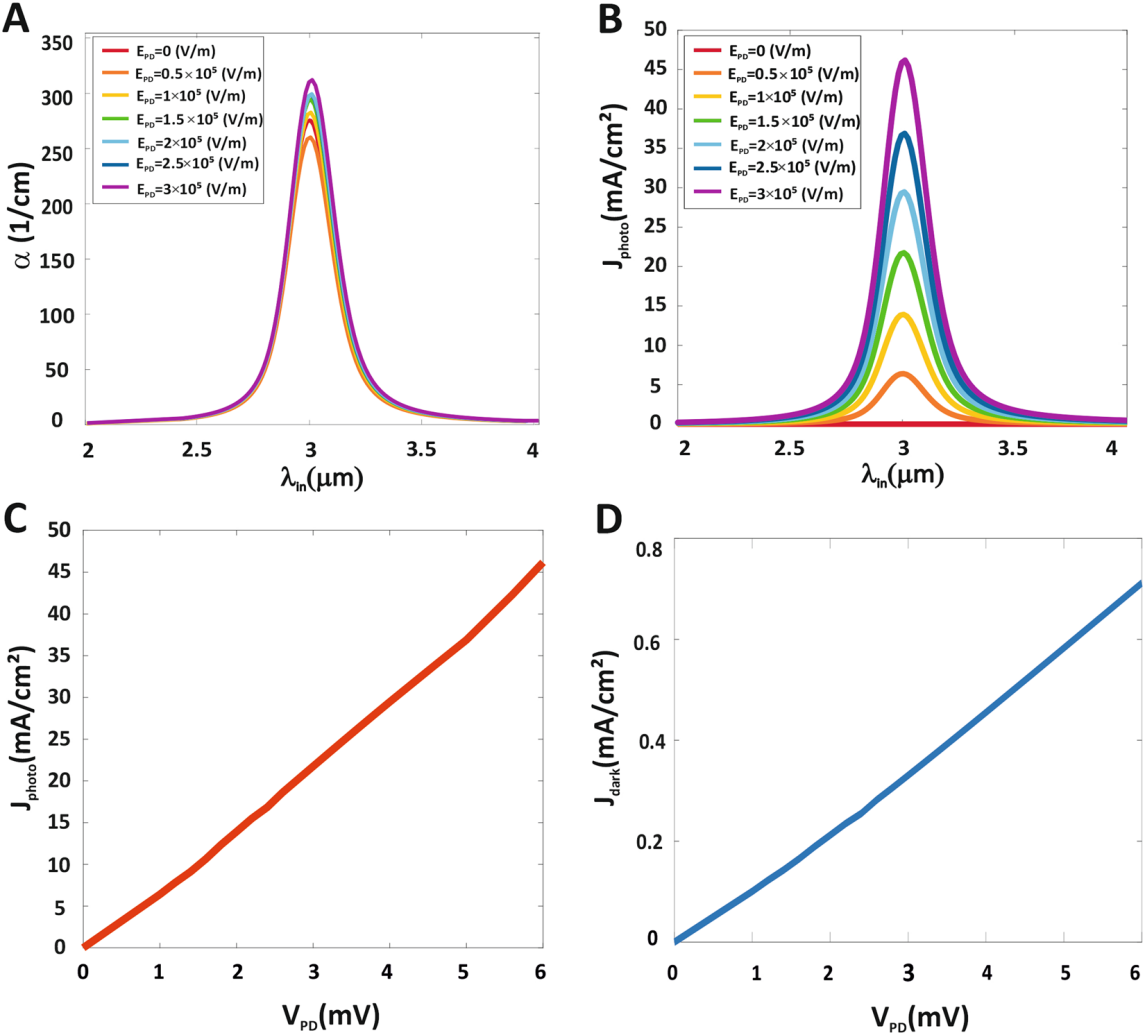
Photocurrent density of MIR QDPD. (**A**) absorption coefficient, (**B**) Photocurrent density of PbSe QDPD as a function of the wavelength under different electrical fields. (**C**) I–V characteristics of the PbSe QDPD under MIR ligth illumination (λ = 3 μm, 0.5 mW/cm^2^) and (**D**) I–V characteristics of the PbSe QDPD in dark.

Figure 2(A) shows the total intersubband absorption coefficient performed at PbSe QDPD as a function of wavelength under different electrical fields. The maximum peak can be easily linked to the sum of intersubband transitions between energy levels of GS and energy levels of ES. The maximum absorption coefficient at 3 μm wavelength and electrical field of 3 × 10^5^ V/m is 312 1/cm. Figure 2(B) shows the QDPD photocurrent density spectrum as a function of under different electrical fields. The peak wavelength in photocurrent density is 3 μm and the maximum photocurrent density at peak wavelength and electrical field of 3 × 10^5^ V/m are 46 mA/cm^2^. The photocurrent density spectrum follows the absorption spectrum of PbSe QDs, that PbSe QDs show a brode wavelength range covering ($3\, \sim \,5\,{\rm{\mu }}{\rm{m}}$). Thus, increasing the electrical field will improve the photocurrent density, but increasing the wavelength of the illumination light from 3 μm results in a decrease in the photocurrent density due to the PbSe QD absorption peak is 3 μm. Figure 2(C,D) shows current density-voltage characteristics of the PbSe QDPD in the dark and under 3 μm MIR light illumination (0.5 mW/cm^2^) whose active area is 20.68 nm × 20.68 nm. The dark current density of QDPD at the voltage of 6 mV is roughly 0.7 mA/cm^2^. Low dark current density is important because it contributes to the Johnson noise in PD. Under MIR light illumination (λ = 3 μm, 0.5 mW/cm^2^), the photocurrent density for a voltage of 6 mV is 46 mA/cm^2^.

**Figure 3:**
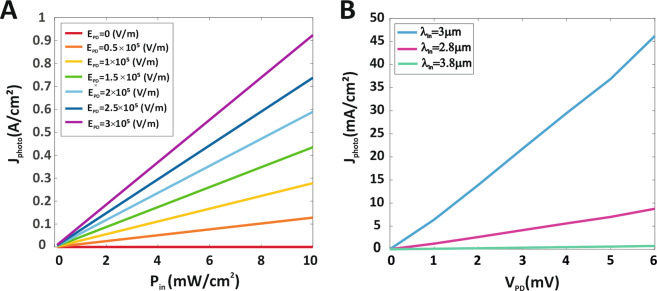
Photocurrent density of MIR QDPD. (**A**) J-P characteristics of PbSe QDPD under different electrical fields (Incident MIR wavelength is 3 μm). (**B**) J-V characteristics of PbSe QDPD under MIR ligth illumination with various wavelengths (Incident MIR power density at different wavelengths is 5 mW/cm^2^).

Figure 3(A) shows the photocurrent-power density characteristics of the PbSe QDPD under a 3 μm MIR light illumination under different electrical fields to comper the effect of the electrical field on PD. As can be seen in Fig. 3(A), the photocurrent increases linearly with increasing radiation light power density due to the strong dependence of the photocurrent on the radiation light power. Figure 3(B) shows the photocurrent density-voltage characteristics of the PbSe QDPD under MIR illumination for three different wavelengths of 2.8, 3, and 3.8 μm with MIR power density 0.5 mW/cm^2^. The photocurrent density of QDPD at a voltage of 6 mV under MIR illumination for three different wavelengths of 2.8, 3, and 3.8 μm is 8.8 mA/cm^2^, 46 mA/cm^2^, 0.71 mA/cm^2^, respectively. Thus the maximum photocurrent density is related to wavelength 3 μm because of the absorption peak of the PbSe QD at that wavelength, and also increasing the wavelength of the illumination light results in a decrease in the photocurrent density.

**Figure 4:**
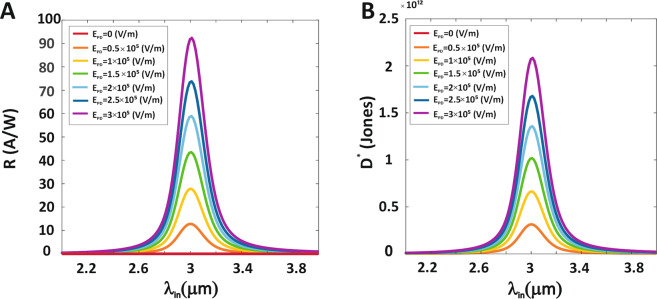
Responsivity and Detectivity spectrums of MIR QDPD. (**A**) Responsivity spectrum, (**B**) Detectivity spectrum of of PbSe QDPD as a function of the wavelength under different electrical fields at 300 K.

To present the best performing PbSe QDPD, responsivity and the detectivity spectrum taken in to account. The responsivity of a PD is known to depend strongly on the photocurrent density in the constant illumination MIR power density. Figure 4(A) illustrates the responsivity spectrum calculated as a function of wavelength under different electrical fields at 300 K for constant illumination MIR power density of 0.5 mW/cm^2^. As a result, the maximum responsivity at peak wavelength (3 μm) is R = 92.37 A/W at the electrical field of 3 × 10^5^ V/m. At 3.8 μm (at the electrical field of 3 × 10^5^ V/m), responsivity is reduced to ~10 A/W because less light is absorbed by the PbSe QDPD. The detectivity spectrum as a function of wavelength under different electrical fields at 300 K for illumination MIR power density of 0.5 mW/cm^2^ is shown in Fig. 4(B). According to Fig. 4(B), the peak detectivity for the response at 3 μm at 300 K and 3 × 10^5^ V/m electrical field is calculated at 2.08 × 10^12^ Jones. The enhancement of the responsivity and the reduction of the dark current improves the detectivity. Thus, the responsivity and the detectivity spectrum increases with an increasing electric field, but increasing the wavelength of the illumination light from 3 μm results in a decrease in the responsivity and the detectivity spectrum due to the PbSe QD absorption peak is 3 μm.

**Figure 5:**
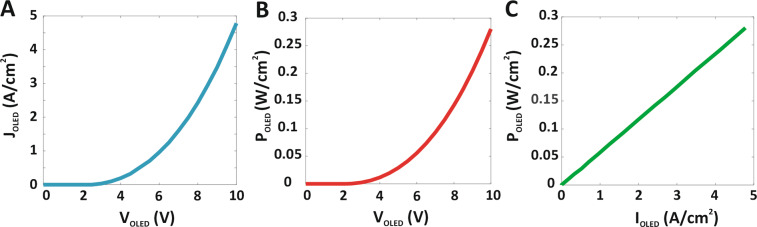
The performance of the OLED. (**A**) J–V, (**B**) P–V and (**C**) P–J charecteristics of OLED.

As shown in Fig. 1(A), photo-generated electrons in the PbSe QD layer are injected into the Transistor collector. These electrons are amplified by a β=100 and this amplified current is injected into the OLED ETL. The recombination of electrons with the distribution of holes from the OLED ITO anode results in visible light. According to the calculations made for the QDPD and the curve shown in Fig. 2, the QDPD photocurrent density is 46 mA/cm^2^. This current is amplified by the Transistor and gives a current of 46 mA/cm^2^ × β = 4.6 A/cm^2^. Figure 5(A,B) show the OLED current density and output power density as a function of the OLED device voltage. Our OLED device behaves like normal OLEDs with a high turn-on voltage. The OLED current density turn on voltage under MIR irradiation is around 5 V. The OLED power density at 10 V is 0.28 W/cm^2^ under an illumination MIR power density of 0.5 mW/cm^2^. Also, Fig. 5(C) shows the OLED output power density as a function of the OLED current density.

**Figure 6:**
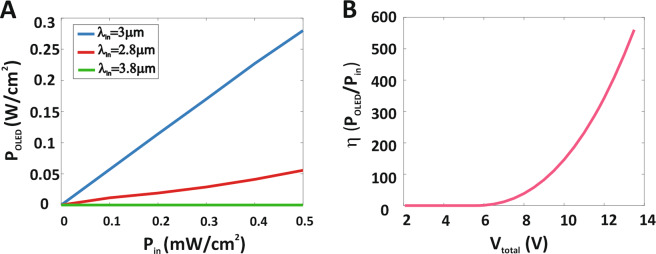
MIR-to-Visible light up-conversion device. (**A**) The Output power density as function of the illumination MIR power density with various wavelengths. (**B**) The efficiency of PbSe up-conversion device as function of the voltage under MIR light illumination (λ = 3 μm, 0.5 mW/cm^2^).

To accurate perception, the output light power density as a function of the illumination MIR power density for the three different wavelengths, 2.8, 3, and 3.8 μm are shown in Fig. 6(A). As shown in Fig. 6(A), the maximum output visible power density for different incoming wavelengths corresponds to a blue diagram with an incident radiation wavelength of 3 μm and this is because of the absorption peak of the PbSe QDPD at that wavelength. Also, the output visible power density increases linearly with increasing radiation light power density. Figure 6(B) shows the up-conversion efficiency as a function of the device total voltage, under 3 μm MIR light illumination (0.5 mW/cm^2^). The EQE^30^ is calculated η = P_out_/P_in_ = 600 for the illumination MIR power density 0.5 mW/cm^2^ under total voltage 13.5 V. Therefore, Our designed up-conversion device has a higher EQE than the previous devices due to PD photocurrent is amplified by the NPN-BJT.

## Conclusion

A new type and high-efficiency integrated optoelectronic chip have been presented to the up-conversion process from MIR to the visible band. In this system, to realize the photoconductive PD, PbSe QDs were used to detect MIR incoming light. Then the output electric current is amplified through NPN-BJT. The amplified electric current was used to drive a green OLED. By applying the MIR signal to the proposed device, a green light is generated by OLED. According to the results, the EQE from MIR to visible is more than (η = P_out_/P_in_ = 600), which is so amazing for the up-conversion process. Also, this device is sensitive to more than 3 μm.
